# Short Report: Social Perception of High School Students with ASD in Norway

**DOI:** 10.1007/s10803-019-04281-w

**Published:** 2019-11-06

**Authors:** Ingjerd Skafle, Anders Nordahl-Hansen, Roald A. Øien

**Affiliations:** 1 Faculty of Education, Østfold University College, B R A Veien 4, P.O. 700, Halden, Norway; 2 Department of Education, UiT – The Arctic University of Norway, 9037 Tromsö, Norway; 3 Child Study Center, Yale University School of Medicine, New Haven, USA

**Keywords:** Autism, Inclusive highs school, Social competence, Qualitative interview, Asperger syndrome, School transition

## Abstract

An increasing number of students with autism spectrum disorder (ASD) enroll in inclusive schools and classrooms. The aim of this study was to research how students with ASD experience the social aspect of inclusive high schools. Five adolescences with Asperger syndrome were interviewed, and the results show that high school was perceived as an important platform for social training, and an equally important place to find new friends and acquaintances. A majority of the participants had experienced loneliness and bullying in junior high school. However, they experienced high school as a new start, with a more open and inclusive environment. Nevertheless, several of the participants expressed that they used quite a lot of energy on social settings, such as interpreting social situations and on being amongst a larger group of students. In order to support this group of adolescents in their schooling, it is important to look at their strength and resources, and not only focus on the challenges and difficulties.

## Introduction

Students with autism spectrum disorder (ASD) without intellectual disabilities (IDs) are at risk of being socially excluded in school-settings, as one of the hallmarks of ASD is to have challenges when it comes to social communication. Students with ASD who fall within the normal range IQ can experience daily misunderstandings, feelings of loneliness and of being different, and difficulties with regards to friendships. However, these challenges can come across as “invisible” to peers and teachers, and thus making them unaware of the daily struggle students with ASD without ID go through. Studies show that students with ASD both with and without ID experience more bullying than their peers (Williams et al. 2017). Many also experience an increased feeling of loneliness and isolation in their teens (Lasgaard et al. 2010). Students with ASD are also at risk of school refusal behavior. A Norwegian study showed that school refusal behavior was significantly higher in students with ASD, aged 9 to 16 years without ID, as compared to typically developing students (TD-students; Munkhaugen et al. 2017). The researchers also suggest that students with normal range IQ and ASD are more at risk of school refusal behavior than students with ID and ASD. Approximately a third of individuals with normal range IQ and ASD experience depression and anxiety (Koegel et al. 2012). Some studies also show that individuals with ASD are at risk of suicide and in particular the group with ASD with a normal range IQ (Richa et al. 2014).

Adolescence requires more social skills and competency compared to earlier age-stages, putting additional stress on students with ASD (White and Roberson-Nay 2009; Øien et al. 2019). In a systematic review by Williams et al. (2017) examining 31 qualitative studies of students with ASD in inclusive schools, three aspects of their school experience contributed to the feeling of being different in a negative way. First, they were self-aware of their diagnosis and that they felt different. Second, the students experienced challenges in being with their peers, and in a large crowd. Third, the physical school environment itself could be challenging, such as noise. These challenges affect students with ASD everyday life at school and beyond, and are bound to interfere in other areas such as academic performance and quality of life at varying degrees for this group of students. Further, there is a concern that mainstream schools may accentuate students with ASD’s mental health problems (Williams et al. 2017).

Norway has adopted the global ideology of inclusion on social and educational policy, meaning that students with ASD are more likely to attend mainstream schools than before (Nilsen 2017; Øien and Nordahl-Hansen 2018). Even though special education schools still exists, there is a political will that most students are attending in inclusive school settings. Students with ASD in Norway may or may not receive special education support depending on their needs (Øien and Nordahl-Hansen 2018). In many instances where the student has an ASD-diagnosis with a normal range IQ, the student does not receive any special education support.

There is an increasing number of students with ASD enrolling in inclusive schools and colleges than ever before (Elias and White 2018), and even though there is an increasing number of papers on the subject, the empirical body of knowledge is still scarce. The purpose of this study is to investigate how students with ASD within normal range IQ experience the social aspect in inclusive high schools. Our specific research questions are as follows; (1) how is school an arena for social interaction, and (2) do students with ASD feel that the schools support their social needs.

## Methods

### Participants

The study was introduced through advertising on the Norwegian Autism Association website, to find students diagnosed with ASD, aged 16 to 18 years old, enrolled in inclusive high schools in the southeastern part of Norway. Three of the participants were recruited via the website. Two participants were recruited through contacting inclusive high schools directly. One female and four males, all diagnosed with Asperger syndrome (AS) agreed to participate in this study. The International Classification of Diseases and Related Health Problems (ICD-10) was still in effect in Norway when this study was undertaken. Thus, the participants have a diagnosis according to the ICD-10 manual. AS is characterized by abnormalities of social interaction and a restricted, stereotyped and repetitive repertoire of interest and activities. AS differs from autism in that there is no general delay in language or cognitive development (World Health Organization 1992). The participants attended inclusive high schools in both rural and urban areas. Questions about co-occurring mental health conditions were not asked, and thus unknown.

### Data Collection

Data was collected through the use of semi-structured individual interviews. All of the participants were offered to see the questions from the interview guide in advance of the interview, but all declined. The interviews took place at a location chosen by each participant. The interviews lasted on average 45 min, with variations from 30 to 60 min, and were recorded on a portable recorder and subsequently transcribed. The interview guide consisted of 12 main questions, which dealt with social aspects of school such as “What does the phrase ‘to be social’ mean to you? Do you have any examples?” or “What does it entail to be social in school?” Besides, examples of social situations from high schools were discussed. The transcriptions sought to reflect the participants’ language as correct and vividly as possible, though not accounting for intonation and pauses as in conversation analysis.

### Ethical Considerations

The Norwegian Centre for Research Data (NSD) approved the project before the study. Consultations with Regional Committees for Medical and Health Research Issues (REK) were also undertaken to ensure that ethical considerations regarding the participants’ diagnosis and age were secured. The participants got written information and a consent form. Because of the students’ age, parents did not need to provide consent. However, due to the participants’ diagnosis, the parents were handed written information about the aim and scope of the study.

### Analysis

The data material was analyzed using a thematic analysis (Braun and Clarke 2006). The initial codes were made up of in vivo-codes, which consist of the participants’ statements (Corbin and Strauss 2008). In this study, 346 initial codes were further analyzed into patterns of themes by the first author. The aim at this stage of the process is to break down the empirical material, yet preserve the essence. In the last stage of the process of using thematic analysis, patterns or themes are decided, moving from the descriptive in vivo-codes to more interpreted and abstract themes (Braun and Clarke 2006). First, each participants’ codes were merged into themes. Then each participants’ themes were analyzed and assembled so that the themes would give an account of the whole set of qualitative data. Although the first and last author had regular meetings to discuss the analysis the first author was responsible for analyzing the raw-data.

## Results

The first step of narrowing in on the essence from the interviews showed that the participants focused on seven key themes: high school as a new start, social challenges, experience of bullying in junior high school, social training at school, most of social activities take place at school, friends and acquaintances, and school as an important social arena.

**Fig. 1:**
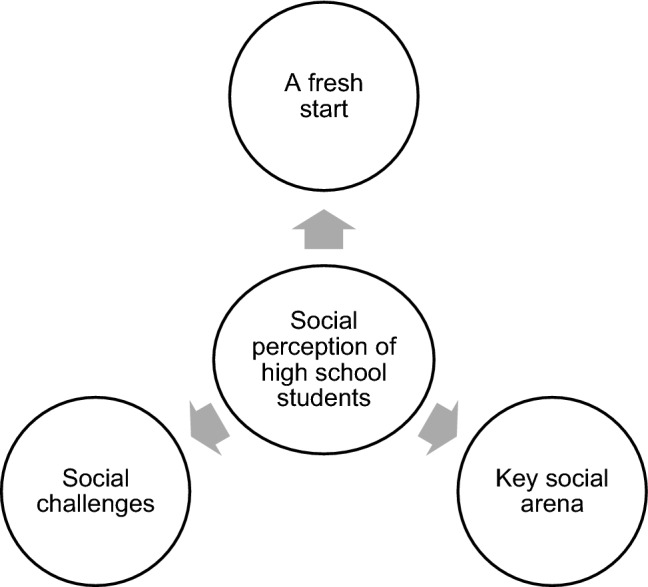
Centre circle illustrate study theme. Arrows indicate substraction of the final process theme selection represented in the three outer circles

In the figure below we present the main themes crystalized from the initial first stage theme selection. The centre circle of the figure indicate the topic that was the target of the study and at the core of the interview-guide. The three main themes surrounding the centre circle are the themes that became prominent through the subsequent thematic analysis of data from the interviews. These were; (1) school is *the* key social arena, (2) transition can be a fresh start, and (3) social challenges (Fig. 1).

### School as a Key Social Arena

The participants explicitly expressed the importance of being social. School was an important social arena where they met new people and made new friendships or acquaintances. Some said that being «forced» to be in a social environment in the school was positive because it gave them a push into the social world. A majority of the participants also expressed that everyday interaction with TD-students increased their social knowledge and that they needed this interaction to learn more about social cues. Social activities in school were also viewed as positive, since being part of the social environment led to natural social training opportunities that the participants took responsibility for themselves. Several participants explained that they actively practiced on being social, and on understanding social codes in school. One of the participants was asked whether high school had made him more social, and he replied:

Yes, talking to people, or being forced to talk to people.. speaking in class and speaking, in general, has helped me a lot. Had I not been given the chance to talk to new people, people with other opinions, my social life would have been destroyed a long time ago.

### A Fresh Start

Junior high school and high school in Norway are separate entities in terms of geographic location and classes are typically made up of completely different students from various schools. Although, a common expectation would be that such transitions would be a negative thing for students with ASD. However, the results from our small sample indicate that this is not always the case. The participants were for the most part positive in their account of inclusive high schools. They seemed to have a good social life and talked positively about their school. Four out of five had experienced bullying or the feeling of being excluded in junior high school and explained that they felt “trapped” in a certain role. Thus, being given a chance to start «fresh» with new students was a positive experience, despite the notion that students with ASD have difficulties with transitions or in being flexible (Brady et al. 2017). It seems as if the feeling of community was enhanced in high school. One of the participants described his high school experience as a “fresh start”:

The nice thing about this school is that there are not that many from my junior high school here. Then I didn’t need to worry about trying to enter social circles already established.. I could find my own. It was so nice, and the main reason why I enrolled in this school.

### Social Challenges

Despite the positive account of high school, the participants talked about some of their challenges. Noise and sound were mentioned as challenging, without being something they wanted support in handling. Crowds seemed to be the most challenging aspect of social life in school. For example, one of the participants worried about whether he should smile to all of his peers during breaks or not: “This is where I feel that the Asperger in me is most striking. When insecurity builds up inside of me. Should I do this? Is this wise? Is this dangerous? Even though it is not dangerous at all». Similar worries were expressed by the other participants and applied to lessons and breaks, and in situations such as where one tries to engage in a conversation without knowing the topic on beforehand. This could cause stress and quite a lot of concern. However, it was also looked upon as something that must be dealt with, and not avoided.

## Discussion

This study elucidates an understanding of the social perception of high school amongst students with ASD. First, the results indicate that school is a very important social arena for the participants, where new friends can be found, and where one can practice on being social. The participants also expressed that everyday interaction with TD-students increase their social knowledge, and also gave opportunities to learn more about being social. A majority of the participants expressed they needed this interaction to be and keep social, but also to learn more about how to interact with others. Interventions aimed to help autistic students often focus on structure and predictability, especially taken into account challenges with regards to executive function and weak central coherence (Happé and Frith 2006; Turner 1999). Even though such interventions can be helpful and sometimes most necessary to prevent stress and confusion, one could argue that cognitive flexibility and social competency are not challenged (Berger et al. 2003).

### A Fresh Start

Many studies focus on important challenges for students with ASD and especially considering transitions between schools (Nuske et al. 2018). This study shows that in addition to the many challenges autistic students face, school transitions can be a new and positive start for some. The students expressed that they felt included, and had positive learning experiences on how to be social, without receiving any type of intervention. Previous research also shows that TD-students can be of good support, and also become role models for ASD-students (Koegel et al. 2012). Students with ASD, however, are at risk of being bullied (Williams et al. 2017). In a recent US-study, 51% out of 35 high school students with high-functioning ASD reported being bullied (Van Schalkwyk et al. 2018).

A majority of the participants in this study explained that they had experienced bullying in junior high school, but none had experienced this in high school. One might, therefore, speculate whether the schools the participants had enrolled in had a student population that suited them well. On the other hand, the schools might have had an inclusive and supportive environment in general.

### Social Challenges

Despite the overall positive account of high school, the participants also talked about social challenges, some more explicit than others. The most challenging aspect is that of interacting with larger groups of students, but also in engaging in conversation that one had not participated in from the beginning. Larger groups of people will increase information-flow and noise. This might increase stress and anxiety in ASD-students, as some have a more detail-oriented cognitive style in line with weak central coherence theory (Happé and Frith 2006). Hence, a larger group of people might come across as complex. It follows then that not being part of a conversation from the beginning might enhance such challenges. Besides, challenges in cognitive flexibility might increase the need for predictability (Brady et al. 2017). A larger group of people is more unpredictable than a smaller group. Despite the social challenges these participants experienced, they seemed determined to face the challenges and learn more about how to handle these situations.

### Limitations

One important limitation for this present study is the selection, which only consists of five participants. Also, the participants were geographically situated in the eastern part of Norway. The generalizability of the findings are restricted and compared to similar research the results of the study could be viewed as an example of the differences in experiences for a group. Another important limitation is the characteristics of the schools, as the participants, by coincidence, were all enrolled in schools with a “good reputation”, and where you need good grades to enter. This may account for some of the positive high school experiences expressed by the participants. Another weakness of the study is that although the first- and third-author worked closely to enhance credibility of the analysis, there was not full double coding of the analysis. The credibility of the data could have been enhanced by the use of more detailed member checks and peer debriefing. A limitation of this study is also a lack of information about concurrent conditions.

### Future Directions

More research is needed about interventions for students with ASD, as an increasing number of students with ASD attend inclusive high schools. Further, more research about the transition to high school need to be undertaken, and also about school life for this group of students in high school in general. Some students with ASD thrive in inclusive classrooms. Others seem to show strength and determination in their quest to learn more about social skills, and in mastering social situations. There is always a need to address the challenges met by autistic people in school. However, flipping the coin and investigating what works will also give key information as to how we go about tailoring interventions and support autistic students in their everyday life at school. Thus, researching the experiences of students with ASD who appear to thrive in a mainstream school environment may be useful to support those who struggle. Furthermore, it is important to investigate factors such as policies and culture that can differ from school to school as well as levels of peers’ understanding of conditions like ASD. This could give important information about optimal environments for students with ASD in inclusive schools.

## Conclusion

The students expressed that it was very important for them to be social and that high school was a significant social arena. In school, they were exposed to an inclusive social setting, something several of the participants noted being necessary for social training. They also made new friends and acquaintances as well as leaving experiences of being bullied behind and feelings of social exclusion. Despite the positive accounts of high school, many used a lot of energy on social situations. To support students with ASD, it is important to create a supportive environment that also acknowledges their strength and resources.
